# Development and initial validation of a brief 2-item measure of belonging among student physical therapists in the United States: a psychometric validation study

**DOI:** 10.3352/jeehp.2026.23.11

**Published:** 2026-05-28

**Authors:** Robyn Redline, Ashanti Jones, Thomas Gus Almonroeder

**Affiliations:** 1 Department of Physical Therapy, School of Health Sciences and Education, Saint Francis University, Loretto, PA, USA; 2 Doctor of Physical Therapy Program, School of Allied Health, College of Health Sciences, University of Louisiana Monroe, Monroe, LA, USA; 3 Brooks College of Health Professions, Trine University, Fort Wayne, IN, USA; Hallym University, Korea

**Keywords:** Students, health occupations, Educational measurement, Psychometrics, Social support, Surveys and questionnaires

## Abstract

**Purpose:**

The objectives of this study were to develop a 2-item abbreviated version of the Program Sense of Belonging questionnaire (ProSBq) and to evaluate its ability to identify student physical therapists with relatively low valued competence and social acceptance.

**Methods:**

A cross-sectional study was conducted using survey data from 634 students enrolled in physical therapist education programs across the United States. The 10-item ProSBq was used to assess 2 dimensions of belonging: valued competence and social acceptance. Principal component analysis was performed to identify representative items for each subscale, with 1 item selected per subscale. Pearson product-moment correlations were used to examine relationships between the single items and their corresponding subscale scores. Classification performance was evaluated by assessing how accurately the single-item responses classified students reporting a relatively low sense of valued competence and social acceptance, based on their full ProSBq subscale scores. Multiple single-item response thresholds were examined to assess classification accuracy.

**Results:**

The single items demonstrated strong relationships with their corresponding subscale scores (r=0.63–0.80, with part-whole correction). For valued competence, sensitivity increased from 53.6% to 92.9%, whereas specificity decreased from 96.3% to 73.5% when a more inclusive threshold was used. A similar sensitivity-specificity tradeoff was observed for social acceptance. Receiver operating characteristic curve analyses demonstrated excellent discrimination (area under the curve ≥0.90).

**Conclusion:**

Single ProSBq items demonstrated strong relationships with full valued competence and social acceptance subscale scores and acceptable classification performance. The abbreviated 2-item ProSBq may provide a practical and efficient method for identifying students experiencing low valued competence or social acceptance.

## Graphical abstract

[Fig f1-jeehp-23-11]

## Introduction

### Background/rationale

In educational contexts, a student’s sense of belonging is defined as feeling accepted, cared for, valued, and respected by peers, faculty, and staff [1]. A sense of belonging can positively influence students’ academic achievement, persistence, and professional identity formation and has also been associated with better mental health and a lower risk of burnout [2-4].

Because a sense of belonging appears to be important for students’ academic success, professional identity formation, and mental health, scales have been developed to measure this construct. Several belongingness scales have been validated for use in health professions education [5-7]. However, the length of these scales, typically 10–40 items, limits their feasibility for routine use, particularly in student advising contexts.

One such scale is the Program Sense of Belonging questionnaire (ProSBq), which was developed to assess belonging among student physical therapists [5,8]. During the initial development of the ProSBq, Almonroeder et al. [5] evaluated its factor structure and identified 2 dimensions of belonging: valued competence and social acceptance. The valued competence subscale reflects students’ perceptions of being respected, valued, and recognized by faculty and staff, whereas the social acceptance subscale reflects students’ perceptions of acceptance and support from peers. These subscales align with established frameworks of student belonging, which emphasize the importance of faculty-student and peer-to-peer relationships [1,4]. In a follow-up study, the 2-factor structure of the ProSBq was confirmed in a separate sample of student physical therapists [8].

The ProSBq includes 10 items, with 5 items per subscale. Although the ProSBq demonstrates acceptable construct validity, an abbreviated version would make the tool easier to implement. Abbreviated assessment tools are commonly used to reduce respondent burden and facilitate the early identification of individuals who may benefit from additional support. For example, 2-item abbreviated measures of depression [9] and burnout [10] have been developed after more comprehensive scales were established to assess these constructs. An abbreviated student belonging scale could make it easier to identify students with relatively low valued competence and/or social acceptance, allowing for earlier and more targeted student support.

### Objectives

The primary objective of this study was to develop an abbreviated version of the ProSBq consisting of a single item for the valued competence subscale and a single item for the social acceptance subscale. A secondary objective was to evaluate the extent to which these single-item responses could accurately classify students reporting relatively low valued competence and/or social acceptance, based on their full ProSBq subscale scores.

## Methods

### Ethics statement

This study involved a secondary analysis of survey data previously used to examine the validity of the ProSBq [8]. The original study protocol was approved by the Institutional Review Board at Northern Illinois University (approval number: HS25-0063). Participation was voluntary, and informed consent was obtained electronically, with survey submission indicating consent to participate. No personal identifiers were collected, and no incentives were provided.

### Study design

This was a development and initial validation study of an abbreviated questionnaire that used secondary cross-sectional survey data from student physical therapists in the United States (Supplement 1).

### Setting

This study was conducted using survey data collected from student physical therapists enrolled in accredited physical therapist education programs across the United States. Participants were recruited via email distribution to program directors and through social media posts to various student special interest groups. Data were collected using an electronic survey administered between October and December 2024.

### Participants

Student physical therapists enrolled in an accredited physical therapist education program in the United States were eligible to participate. There were no specific exclusion criteria beyond incomplete ProSBq responses. All physical therapist education programs in the United States are at the doctoral level.

### Variables

The variables of interest were responses to the 10 ProSBq items. The ProSBq includes valued competence (items 1–5) and social acceptance (items 6–10) subscales.

### Data sources/measurement

Data were obtained from an electronic survey administered via Qualtrics (https://www.qualtrics.com/). The survey included the full 10-item ProSBq, which was developed to assess a sense of belonging among student physical therapists and has demonstrated acceptable construct validity and a stable 2-factor structure, comprising valued competence and social acceptance, in prior studies [5,8]. Each item is rated on a 6-point Likert scale ranging from strongly disagree to strongly agree. The ProSBq was developed by the study authors and is provided in Supplement 2.

### Bias

Study participation was voluntary, which may have introduced self-selection bias, as students with a particularly high or low sense of belonging may have been more likely to respond. In addition, recruitment through program directors and social media may have resulted in uneven participation across programs or regions, although efforts were made to recruit from a broad range of programs across the United States. The survey was anonymous to reduce social desirability bias.

### Study size

This study involved a secondary analysis of an existing dataset used to examine the construct validity of the ProSBq [8]. To limit optimism bias, the dataset was randomly divided into development (70% of the sample, n=444) and validation (30% of the sample, n=190) samples. Item identification was conducted using the development sample, whereas classification performance was evaluated with the independent validation sample. Principal component analysis was used for item identification. With 10 items, the development sample of 444 participants provided more than 40 observations per item, substantially exceeding commonly cited subject-to-item ratio guidelines for principal component analysis, such as 10:1 [11], and supporting the stability of the component solution.

### Statistical methods

Likert scale responses were transformed to numeric values for analysis: strongly disagree=1, disagree=2, slightly disagree=3, slightly agree=4, agree=5, and strongly agree=6. Principal component analysis was conducted with the development sample data (n=444) to extract components representing the primary sources of variance in the ProSBq responses based on the inter-item correlation matrix [12]. Component loadings reflect the correlation between each item and the retained component, with higher absolute values indicating stronger associations. Parallel analysis was conducted to determine the number of components to retain by comparing observed eigenvalues with those generated from random data [13]. An oblique oblimin rotation was applied to aid interpretation of the component structure. Oblique rotation was selected because prior validation studies [5,8] demonstrated that the valued competence and social acceptance subscales are moderately correlated. Oblique rotation methods are recommended when components are correlated [12]. The loadings associated with each retained component were examined, and the item with the highest absolute loading on each component was identified as the candidate single item for the valued competence and social acceptance subscales. This approach has previously been used to identify suitable items when developing abbreviated measures [10]. All other analyses, including correlation analyses and classification accuracy analyses, were conducted using the validation sample data (n=190).

Pearson product-moment correlations were used to evaluate the relationships between each single item and the mean score of its corresponding subscale. Correlations were analyzed between the item and (1) the full subscale mean, including the item, and (2) the subscale mean with the item removed to reduce part-whole inflation [10]. The strength of the relationships was interpreted based on the magnitude of the correlation coefficient (r), using the following guidelines: 0.20–0.39=weak, 0.40–0.59=moderate, 0.60–0.79=strong, and ≥0.80=very strong [14]. Ninety-five percent confidence intervals (95% CIs) were calculated for each correlation coefficient. The coefficient of determination (r^2^) was used to describe the proportion of variance in the subscale mean explained by the single item.

A secondary objective was to determine whether the selected items could identify students exhibiting relatively low valued competence and/or social acceptance. Students were classified as having low valued competence and/or social acceptance if their mean subscale score across all 5 items was less than 4.0. On the 6-point Likert scale used in the ProSBq, a score of 4 corresponds to “slightly agree.” Therefore, a mean score <4.0 indicates responses that, on average, do not reflect even slight agreement with positively worded statements related to valued competence or social acceptance. This threshold was selected to identify students who generally do not report at least slight agreement and may warrant follow-up, rather than to reflect the statistical midpoint of the scale, such as 3.5. Single-item performance was evaluated using 2 thresholds. First, an item response <4 was used to classify students as low on the corresponding construct. In addition, a more inclusive threshold of <5 was examined to determine whether classifying students who did not respond at least “agree” would improve sensitivity for detecting those with low subscale scores, defined as a mean score <4.0. Notably, the subscale classification threshold remained constant across analyses; only the single-item threshold varied, reflecting its intended use to identify students who may need additional support rather than to serve as a direct substitute for the full ProSBq. Sensitivity, specificity, positive predictive values, and negative predictive values were calculated to quantify the ability of each item threshold, <4 and <5, to correctly classify students with relatively low valued competence or social acceptance, defined as a mean subscale score <4.0, and those with acceptable levels, defined as a mean subscale score ≥4.0. Receiver operating characteristic (ROC) curve analyses were conducted to evaluate the overall discriminative ability of the single items for identifying students with relatively low valued competence and social acceptance. The area under the curve (AUC) was calculated to summarize classification performance. In addition, the empirically optimal cutoff was identified using Youden’s index, and the corresponding threshold values were compared with the prespecified <4 and <5 single-item thresholds. Analyses were conducted using JASP ver. 0.19.2.0 (JASP Team) and IBM SPSS ver. 29.0.2.0 (IBM Corp.).

## Results

### Participants

A total of 634 student physical therapists completed the survey (development sample: n=444; validation sample: n=190). Participant characteristics are presented in Table 1.

### Main results

Parallel analysis indicated that 2 components should be retained, as the observed eigenvalues for the first 2 components exceeded the 95th percentile of eigenvalues obtained from randomly generated data, whereas subsequent components did not. These 2 components explained 62.6% of the total variance in the dataset (Component 1=48.1%, Component 2=14.5%). Component 1 was primarily associated with social acceptance items, with higher loadings for items 6–10, whereas Component 2 was primarily associated with valued competence items, with higher loadings for items 1–5 (Table 2). No item demonstrated cross-loadings ≥0.40 on both components, indicating a clear and interpretable simple structure. The 2 components were moderately correlated (r=0.49), supporting the use of oblique rotation. Based on the loading patterns, Item 2 exhibited the highest loading for the valued competence component (loading=0.83), and Item 7 exhibited the highest loading for the social acceptance component (loading=0.90) (Table 2). Therefore, these items were selected as the candidate single items for the valued competence and social acceptance subscales. Item 2, representing valued competence, stated: “Faculty and staff in the physical therapy program value my opinions.” Item 7, representing social acceptance, stated: “I have a good relationship with other students in the physical therapy program.” Dataset 1 includes the 10-item ProSBq responses.

Item 2 demonstrated a strong correlation with its corresponding valued competence subscale score (r=0.78; 95% CI, 0.72–0.83), explaining 61% of the variance in the full subscale mean when all items were included. When Item 2 was omitted from the subscale mean calculation, the relationship remained strong (r=0.63; 95% CI, 0.54–0.71), explaining 40% of the variance in the remaining valued competence items. Similarly, Item 7 demonstrated a very strong correlation with its corresponding social acceptance subscale score (r=0.87; 95% CI, 0.83–0.90), explaining 76% of the variance in the full subscale mean. When Item 7 was omitted from the subscale mean, the relationship remained very strong (r=0.80; 95% CI, 0.74–0.85), explaining 64% of the variance in the remaining social acceptance items.

For valued competence, overall classification accuracy for low (mean subscale score <4.0) versus acceptable (mean subscale score ≥4.0) status was 90.0% when a single-item threshold of <4 was used (Table 3). At this threshold, sensitivity was 53.6%, and specificity was 96.3%. When a more inclusive single-item threshold of <5 was applied, overall accuracy decreased to 76.3%, driven primarily by a reduction in specificity to 73.5%; however, sensitivity increased to 92.9% (Table 3). ROC curve analysis demonstrated excellent discrimination for the valued competence item (AUC=0.90; 95% CI, 0.84–0.95). The empirically optimal cutoff corresponded to a value of approximately 4.5 based on Youden’s index, aligning with a <5 threshold and providing a more favorable balance between sensitivity and specificity than the <4 threshold.

For social acceptance, overall classification accuracy was 90.0% when a single-item threshold of <4 was used (Table 4). At this threshold, sensitivity was 48.5%, and specificity was 98.7%. When the more inclusive single-item threshold of <5 was applied, overall accuracy decreased to 86.8%, with specificity decreasing to 86.0% and sensitivity increasing to 90.9% (Table 4). ROC curve analysis similarly demonstrated excellent discrimination for the social acceptance item (AUC=0.93; 95% CI, 0.89–0.98). The empirically optimal cutoff corresponded to a value of approximately 4.5, aligning with a <5 threshold and providing a more favorable balance between sensitivity and specificity than the <4 threshold.

## Discussion

### Key results

The primary objective of this study was to develop an abbreviated version of the ProSBq consisting of a single item for the valued competence subscale and a single item for the social acceptance subscale. The selected items demonstrated strong correlations with their corresponding subscale scores, indicating that they captured a substantial portion of the variance in each dimension of belonging. A secondary objective was to evaluate the extent to which these single-item responses could accurately classify students reporting relatively low valued competence and/or social acceptance, based on their full ProSBq subscale scores. When classification performance was evaluated, a more inclusive threshold of <5, corresponding to less than “agree,” improved sensitivity and allowed the single items to identify most students with low valued competence and social acceptance. This more inclusive threshold was also better aligned with the empirically optimal cutoff identified through ROC curve analyses.

### Interpretation

These findings suggest that single-item indicators may capture key aspects of belonging, allowing for efficient identification of students who may be experiencing relatively low valued competence and/or social acceptance. A <5 threshold, corresponding to less than “agree,” may be particularly useful in contexts where minimizing missed cases is a priority, although this approach reduces specificity.

The valued competence ProSBq subscale aligns with Strayhorn’s framework of student belonging, which emphasizes the importance of students feeling valued, respected, and supported by faculty and staff [1]. Within health professions education, these experiences are closely tied to professional identity formation, as students integrate their developing skills with their emerging roles as clinicians [3]. When students perceive that their contributions are recognized and that they are cared for within their program, they may develop greater confidence in their abilities and a stronger sense of competence.

The social acceptance ProSBq subscale reflects students’ sense of connection and inclusion among peers, which is a critical component of learning and academic achievement in health professions education [1]. Training in health professions programs inherently relies on social interaction, including formal peer-assisted learning and informal collaborative experiences that support clinical reasoning and skill development [15].

### Comparison with previous studies

The present findings are consistent with prior studies demonstrating that abbreviated instruments can efficiently assess complex constructs such as burnout and depression [9,10]. Similar to these prior studies, the selected ProSBq items showed strong associations with their full subscales, supporting their potential use for identifying students who may benefit from additional support. To our knowledge, no existing abbreviated scale assesses key aspects of student belonging.

### Limitations

The classification approach used in this study included the single item within the subscale mean used to define “low” status, which may introduce some part-whole dependence. This approach is consistent with prior abbreviated scale development studies (e.g., [10]) and was used to maintain the established full ProSBq subscales. However, reducing each subscale to a single item may limit the ability to fully capture the complexity of valued competence and social acceptance. Therefore, the abbreviated ProSBq should not be viewed as a replacement for the full ProSBq. Valued competence, social acceptance, and belonging more broadly are multifaceted constructs that require multiple items to fully capture. For research or comprehensive program assessment purposes, the full ProSBq is likely more appropriate. Finally, although classification performance was evaluated in an independent validation sample, additional external validation in independent cohorts would further strengthen the evidence supporting use of the abbreviated ProSBq.

### Generalizability

Participants were recruited from physical therapist education programs across the United States. However, additional research is needed to determine whether these findings apply to students in other health professions programs or to students outside the United States.

### Suggestions

Prior research indicates that sense of belonging is critical for academic success, mental health, and professional identity formation among health professions students across multiple programs and countries, suggesting that belonging is a fundamental component of learning and professional development [2-4]. Therefore, future research should evaluate the performance of the ProSBq, in both its full and abbreviated forms, in other health professions student populations. Future studies could also apply item response theory or Rasch modeling approaches to evaluate item-level discrimination and information across levels of the underlying construct, which may further refine item selection and enhance the precision of the ProSBq. In practice, the 2-item ProSBq may be administered before advising sessions. Responses below “agree” (<5) on either item may prompt further discussion to explore potential barriers related to valued competence or social acceptance. This approach provides a simple and efficient way to identify students who may benefit from additional support without adding substantial burden to existing workflows.

### Conclusion

A 2-item version of the ProSBq demonstrated strong relationships with the full ProSBq subscale scores and acceptable performance for identifying students with relatively low valued competence and social acceptance. The abbreviated ProSBq may provide a practical and efficient tool for identifying students who warrant additional follow-up.

## Figures and Tables

**Figure f1-jeehp-23-11:**
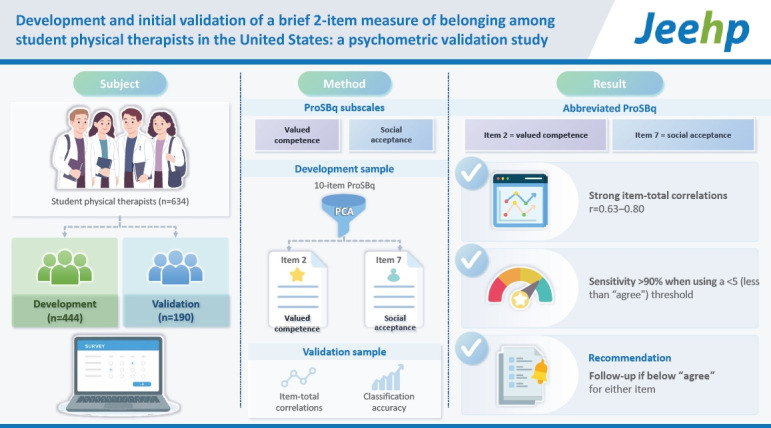


**Table 1.** Characteristics of the sample of student physical therapists (n=634)

Characteristic	Value
Age (yr)	25.34±3.88
Gender identity	
Cisgender female	362 (57.1)
Cisgender male	210 (33.1)
Non-binary	16 (2.5)
Transgender female	6 (0.9)
Transgender male	4 (0.6)
Queer	4 (0.6)
Preferred not to answer	32 (5.0)
Race and ethnicity	
White	292 (46.1)
Black	200 (31.5)
Hispanic/Latinx	31 (4.9)
Asian	29 (4.6)
Middle Eastern	11 (1.7)
Indigenous	7 (1.1)
Multiracial	64 (10.1)
Year in the program	
First year	163 (25.7)
Second year	343 (54.1)
Third year	128 (20.2)
Program format	
Residential program	482 (76.0)
Hybrid program	145 (22.9)
Unsure	7 (1.1)

Values are presented as mean±standard deviation or number (%).

**Table 2.** Principal component loadings and eigenvalues for the Program Sense of Belonging questionnaire

	Valued competence	Social acceptance
Item 1 (VC)	0.59	
Item 2 (VC)	0.83^a)^	
Item 3 (VC)	0.77	
Item 4 (VC)	0.71	
Item 5 (VC)	0.82	
Item 6 (SA)		0.81
Item 7 (SA)		0.90^a)^
Item 8 (SA)		0.78
Item 9 (SA)		0.67
Item 10 (SA)		0.82
Eigenvalues	1.45	4.81

Oblimin rotation applied. Loadings <0.40 not displayed. VC, valued competence; SA, social acceptance.

a) Item selected for abbreviated version (highest component loading).

**Table 3.** Single-item classification performance for the valued competence subscale

	Total	Item ≥4 (acceptable)	Item <4 (low)	Item ≥5 (acceptable)	Item <5 (low)
Subscale mean ≥4.0 (acceptable)	162	156	6	119	43
Subscale mean <4.0 (low)	28	13	15	2	26
Total	190	169	21	121	69
Sensitivity (%)		53.6		92.9	
Specificity (%)		96.3		73.5	
PPV (%)		71.4		37.7	
NPV (%)		92.3		98.3	

Contingency tables with subscale mean classification (<4.0=“low”) in rows and single-item classification in the columns (<4 [top] or <5 [bottom]=“low”). PPV, positive predictive value; NPV, negative predictive value.

**Table 4.** Single-item classification performance for the social acceptance subscale

	Total	Item ≥4 (acceptable)	Item <4 (low)	Item ≥5 (acceptable)	Item <5 (low)
Subscale mean ≥4.0 (acceptable)	157	155	2	135	22
Subscale mean <4.0 (low)	33	17	16	3	30
Total	190	172	18	138	52
Sensitivity (%)		48.5		90.9	
Specificity (%)		98.7		86.0	
PPV (%)		88.9		57.7	
NPV (%)		90.1		97.8	

Contingency tables with subscale mean classification (<4.0=“low”) in rows and single-item classification in the columns (<4 [top] or <5 [bottom]=“low”). PPV, positive predictive value; NPV, negative predictive value.

## Supplementary materials

Supplement 1. STROBE Statement—checklist of items that should be included in reports of observational studies.

Supplement 2. 10-item version of the ProSBq.

Supplement 3. Audio recording of the abstract.
