# Supplementary material for: Development and initial validation of a brief 2-item measure of belonging among student physical therapists in the United States: a psychometric validation study
Source: J Educ Eval Health Prof. 2026 May 28;23:11. doi: 10.3352/jeehp.2026.23.11 (PMC13276327; doi:10.3352/jeehp.2026.23.11)
Supplement: Supplementary file 3 — Supplement 2. 10-item version of the ProSBq. [file jeehp-23-11-suppl2.docx]

**Program Sense of Belonging (ProSB) Questionnaire**

Please rate the extent to which you agree with the following statements:

**Valued Competence**

1. People in the physical therapy program notice when I am good at something.

 strongly disagree  disagree  slightly disagree  slightly agree  agree  strongly agree

1. Faculty and staff in the physical therapy program value my opinions.

 strongly disagree  disagree  slightly disagree  slightly agree  agree  strongly agree

1. Most faculty and staff in the physical therapy program are interested in me.

 strongly disagree  disagree  slightly disagree  slightly agree  agree  strongly agree

1. People in the physical therapy program know I can do good work.

 strongly disagree  disagree  slightly disagree  slightly agree  agree  strongly agree

1. The instructors in the physical therapy program give me compliments when I do something good.

 strongly disagree  disagree  slightly disagree  slightly agree  agree  strongly agree

**Social Acceptance**

1. Students in the physical therapy program help each other to succeed.

 strongly disagree  disagree  slightly disagree  slightly agree  agree  strongly agree

1. I have a good relationship with other students in the physical therapy program.

 strongly disagree  disagree  slightly disagree  slightly agree  agree  strongly agree

1. I can really be myself in the physical therapy program.

 strongly disagree  disagree  slightly disagree  slightly agree  agree  strongly agree

1. I feel proud of belonging to the physical therapy program.

 strongly disagree  disagree  slightly disagree  slightly agree  agree  strongly agree

1. Other students in the physical therapy program like me the way I am.

 strongly disagree  disagree  slightly disagree  slightly agree  agree  strongly agree
